# The Morphogenesis, Pathogenesis, and Molecular Regulation of Human Tooth Development—A Histological Review

**DOI:** 10.3390/ijms26136209

**Published:** 2025-06-27

**Authors:** Dorin Novacescu, Cristina Stefania Dumitru, Flavia Zara, Marius Raica, Cristian Silviu Suciu, Alina Cristina Barb, Marina Rakitovan, Antonia Armega Anghelescu, Alexandu Cristian Cindrea, Szekely Diana, Pusa Nela Gaje

**Affiliations:** 1 Department II, Discipline of Histology, Victor Babes University of Medicine and Pharmacy Timisoara, E. Murgu Square, No. 2, 300041 Timisoara, Romania; novacescu.dorin@umft.ro (D.N.); flavia.zara@umft.ro (F.Z.); marius.raica@umft.ro (M.R.); cristian_suciu@umft.ro (C.S.S.); toma.alina@umft.ro (A.C.B.); marina.rakitovan@umft.ro (M.R.); antonia.armega@umft.ro (A.A.A.); alexandru.cindrea@umft.ro (A.C.C.); gaje.nela@umft.ro (P.N.G.); 2 Doctoral School, Victor Babes University of Medicine and Pharmacy Timisoara, E. Murgu Square, No. 2, 300041 Timisoara, Romania; diana.szekely@umft.ro

**Keywords:** odontogenesis, enamel organ, tooth development stages, epithelial–mesenchymal interactions, dental lamina, inner enamel epithelium, dental papilla, morphogenesis, histological analysis

## Abstract

Odontogenesis, the development of teeth, is a complex, multistage process that unfolds from early embryogenesis through tooth eruption and maturation. It serves as a classical model of organogenesis due to the intricate reciprocal interactions between cranial neural crest-derived mesenchyme and oral epithelium. This narrative review synthesizes current scientific knowledge on human tooth development, tracing the journey from the embryological origins in the first branchial arch to the formation of a fully functional tooth and its supporting structures. Key morphogenetic stages—bud, cap, bell, apposition, and root formation—are described in detail, highlighting the cellular events and histological features characterizing each stage. We discuss the molecular and cellular regulatory networks that orchestrate odontogenesis, including the conserved signaling pathways (Wnt, BMP, FGF, SHH, EDA) and transcription factors (e.g., PAX9, MSX1/2, PITX2) that drive tissue patterning and cell differentiation. The coordinated development of supporting periodontal tissues (cementum, periodontal ligament, alveolar bone, gingiva) is also examined as an integral part of tooth organogenesis. Finally, developmental anomalies (such as variations in tooth number, size, and form) and the fate of residual embryonic epithelial cells are reviewed to underscore the clinical significance of developmental processes. Understanding the normal course of odontogenesis provides crucial insight into congenital dental disorders and lays a foundation for advances in regenerative dental medicine.

## 1. Introduction

Tooth development (odontogenesis) is a tightly regulated sequence of events in which embryonic cells interact to form one of the most mineralized structures in the human body. Mammalian tooth formation proceeds through sequential and reciprocal interactions between the oral epithelium (ectoderm) and the underlying cranial neural crest-derived ectomesenchyme [1,2]. These epithelial–mesenchymal interactions are the hallmark of odontogenesis, governing all major steps from initiation of the tooth germ to the morphogenesis of mineralized tissues via an intricate series of inductive events [2]. In humans, odontogenesis begins early in prenatal development: the primary (deciduous) teeth start to form between the 6th and 8th weeks of gestation, and the first permanent teeth buds appear around the 20th week in utero [3]. If the initiation of tooth development does not occur on schedule during these critical periods, the affected teeth may fail to develop and never form at all, resulting in the congenital absence of those teeth (hypodontia or anodontia) [4].

Teeth originate from the first pharyngeal (branchial) arch, where migrating neural crest cells populate the jaw primordia and form ectomesenchyme [5]. The primary epithelial band (primitive oral epithelium) appears around day 37 of embryonic development and marks the first morphological sign of odontogenesis. This band of oral epithelium invaginates into the jaw ectomesenchyme as the dental lamina, which is genetically programmed to initiate tooth formation at specific sites. The dental lamina gives rise to 20 tooth buds for the primary dentition and also produces secondary epithelial outgrowths (successional laminae) that will form the buds of permanent successors (incisors, canines, and premolars). The permanent molars, which have no primary predecessors, originate directly from posterior extensions of the primary dental lamina in the developing jaws [6]. Through these processes, a total of 32 permanent tooth germs are established.

Odontogenesis is typically divided into defined stages—commonly termed the bud, cap, bell, and root formation stages—based on the evolving morphology of the tooth germ. Each stage reflects a continuum of cellular proliferation, differentiation, and tissue patterning events [7]. During the early stages, the oral epithelium is the driving force, releasing growth factors that condense and instruct the underlying mesenchyme. As development progresses, the “dialogue” continues, yet a switch occurs, with the mesenchymal tissue now feeding back signals that will guide epithelial morphogenesis. This stepwise interplay creates the complex structure of a tooth, which is composed of enamel (an epithelial product), dentin (a mesenchymal product), and dental pulp, as well as cementum, periodontal ligament (PDL), and alveolar bone as supporting tissues.

The study of tooth development has broader significance beyond dentistry. The tooth is an excellent model system in developmental biology for unraveling fundamental mechanisms of organogenesis [8]. Insights from tooth morphogenesis and the identification of key signaling networks have informed our understanding of other ectodermal organs and craniofacial developmental disorders. Furthermore, unraveling the molecular controls of odontogenesis paves the way for innovative approaches in tissue engineering and regenerative medicine [8]. For instance, knowledge of the stem cell niches and signaling pathways in developing teeth could eventually enable bioengineered tooth replacement therapies. This review provides a comprehensive overview of human odontogenesis, from embryonic initiation to tooth eruption, and integrates up-to-date findings on molecular regulation. In addition, we highlight how deviations in these developmental processes lead to dental anomalies and the presence of epithelial remnants that may have clinical implications.

Despite the numerous reviews describing specific aspects of odontogenesis, few provide a comprehensive and integrative histological perspective aligned with the dynamics of molecular signaling at all developmental stages. This review offers a unique contribution by systematically correlating morphological transitions (as visualized in the original human histological sections). In addition, we provide an up-to-date synthesis of enamel organ differentiation, the emergence of structural complexity, and the spatial organization of the pulp and supporting tissues. By integrating classical and modern perspectives, this review aims to serve as both a scientific reference and an educational tool for understanding normal dental development and its pathologic deviations.

## 2. Embryology and Conceptual Overview

Odontogenesis is a multifaceted developmental process that entails morphogenesis, epithelial–mesenchymal interactions, fibrillogenesis, and mineralization, culminating in the formation and maturation of a functional tooth germ [9]. The oral cavity originates during the fourth week of embryonic development, as the primitive stomodeum begins to take shape beneath the developing forebrain. At this stage, the cephalic region of the embryo is organized into a series of mesenchymal condensations known as pharyngeal (branchial) arches. In humans, the first and second pharyngeal arches are particularly well-developed and give rise to the maxillary and mandibular prominences, which will ultimately contribute to jaw and tooth formation [10].

The primitive oral cavity, known as the stomodeum, arises as an ectodermal invagination in the ventral region of the developing head during the fifth embryonic week. Around embryonic day 37, a horseshoe-shaped thickening of the oral epithelium—referred to as the primary epithelial band—appears along the future dental arches. This epithelium overlays a population of neural crest-derived mesenchymal cells, termed ectomesenchyme, which plays a pivotal role in craniofacial and dental development. Histologically, the primary epithelial band is composed of 2–3 layers of cuboidal or polygonal epithelial cells, while the underlying ectomesenchymal cells exhibit a stellate morphology and form an interconnected network embedded in a gelatinous extracellular matrix [11].

Humans are diphyodont, developing two successive sets of teeth during their lifetime. The primary dentition (deciduous teeth) typically begins to erupt by approximately 6 months of age and is usually completed by 2.5 years. This is later replaced by the permanent dentition, which begins with the eruption of the first molars around age 6 and continues until the third molars (wisdom teeth) emerge in late adolescence. The permanent dentition comprises two distinct tooth categories: successional teeth—including incisors, canines, and premolars—that replace their primary predecessors; and accessional teeth—the three permanent molars—which develop independently and have no deciduous precursors [12].

The successional dental lamina is a lingual epithelial outgrowth of the primary dental lamina that appears during the 3rd to 4th months of intrauterine life and gives rise to permanent successor teeth—namely, the incisors, canines, and premolars. This structure remains active for an extended developmental window, generating successive buds from the lingual side of each deciduous tooth germ, with the incisors and canines initiating around the 5th prenatal month and the second premolars around the 10th month of gestation [13].

In contrast, the permanent molars (first, second, and third molars), which do not replace any primary teeth, develop directly from posterior extensions of the primary dental lamina and are therefore classified as accessional teeth in the international literature [14].

The initiation of tooth bud development follows a precise chronological sequence. The earliest dental structures to emerge are the buds of the mandibular central incisors, which initiate development during the 7th week of gestation, followed by the maxillary incisors in the 8th week. The canine tooth germs appear shortly thereafter, while the first primary molars begin forming between the 8th and 9th weeks and the second primary molars between the 10th and 11th weeks [15].

In contrast, the permanent molars, which are classified as accessional teeth due to their lack of deciduous predecessors, develop as posterior extensions of the primary dental lamina. The first permanent molars begin to form around the 4th month of intrauterine life, the second molars during the first postnatal year, and the third molars (wisdom teeth) typically emerge after the age of 3 years [16,17].

**Figure 1:**
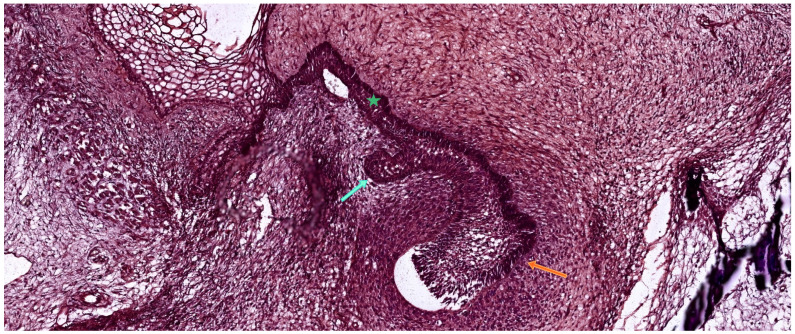
Histological section of human embryonic mandible stained with hematoxylin-eosin (HE), showing two adjacent developing tooth germs. The orange arrow indicates an early cap-stage deciduous tooth germ, while the cyan arrow marks a bud-stage permanent tooth germ derived from the successional dental lamina. The green asterisk identifies the primary dental lamina connecting the developing structures to the oral epithelium. Scale bar = 100 µm. Original magnification: 10×.

As illustrated in Figure 1, the continuity of the primary dental lamina gradually degenerates, leaving behind discrete epithelial segments that correspond to individual tooth germs. These germs become embedded within the surrounding ectomesenchyme, where they continue their development within a dedicated osseous crypt.

The dental lamina remains active over an extended developmental window, spanning approximately five years. During this period, it sequentially gives rise to both primary and permanent tooth germs. The deciduous tooth buds begin to form by the second month of intrauterine life [18]. In parallel, the permanent molars, which have no deciduous precursors, arise from posterior extensions of the primary dental lamina. The first permanent molar initiates development around the 4th month in utero, the second permanent molar during the first postnatal year, and the third molar (wisdom tooth) typically appears between the ages of 4 and 5 years. Simultaneously, another epithelial structure—the vestibular lamina—emerges from the buccal side of the primary epithelial band. Through a process of cellular proliferation and degeneration, it contributes to the formation of the oral vestibule, the anatomical space between the cheeks, lips, and dental arches [16].

A detailed summary of the chronological timeline of both primary and permanent tooth development, along with their embryological origins, is presented in Table 1. This comparative overview highlights the temporal and anatomical distinctions between successional and accessional teeth, as well as the sequential appearance of dental structures throughout pre- and postnatal development [17].

Each dental bud progresses as a distinct anatomical unit, following a highly conserved sequence of morphogenetic and differentiation events. Odontogenesis, the process of dental organ development, occurs in a similar manner for all teeth and involves the orchestrated differentiation of specialized cell types [19]. Within the developing tooth germ, odontoblasts and ameloblasts initiate the formation and mineralization of dentin and enamel, respectively. Subsequently, cementoblasts, periodontal fibroblasts, and osteoblasts contribute to the development of the root, periodontal ligament, and alveolar bone. Root formation and tissue maturation continue until the tooth is structurally complete and functionally integrated into the oral cavity. The tooth eruption process itself is complex, involving coordinated remodeling of surrounding tissues and progressive elongation of the root, which becomes anchored via the periodontal ligament into the adjacent cementum and alveolar bone [17].

The initiation of odontogenesis is driven by reciprocal epithelial–mesenchymal interactions, primarily orchestrated by the dental epithelium derived from the oral ectoderm. These epithelial cells release a complex array of signaling molecules—including growth factors, morphogens, and adhesion proteins—that act upon the underlying neural crest-derived ectomesenchyme, triggering the formation of the tooth germ. The developing tooth germ comprises three key structural components:

(i) the enamel organ, an epithelial structure responsible for enamel production;
(ii) the dental papilla, a condensed mesenchymal core that will give rise to dentin and pulp; and
(iii) the dental follicle (or sac), a surrounding mesenchymal sheath that will form the periodontium, including cementum, periodontal ligament, and alveolar bone [17].

Among these, the enamel organ is the first to become morphologically distinct and serves as the architectural and inductive center of early tooth development.

## 3. Odontogenesis—Developing Proper Dental Tissues

**Figure 2:**
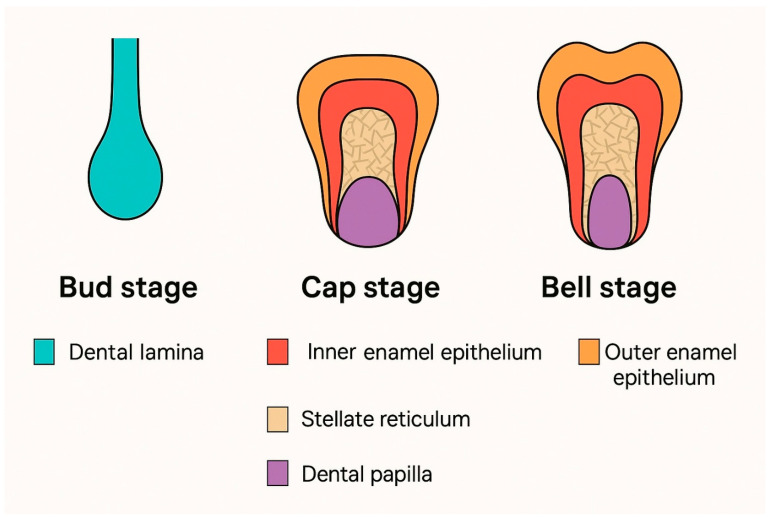
Schematic representation of the main stages of human tooth development: bud stage, cap stage, and bell stage. Key anatomical structures are color-coded for clarity, including the dental lamina, enamel organ epithelium (outer and inner layers), stellate reticulum, and dental papilla.

Following initiation, tooth development proceeds through a well-defined series of morphological stages that reflect both the evolving shape of the enamel organ and the progressive differentiation of the underlying mesenchyme. Although these stages represent a continuous developmental continuum, they are classically categorized as the bud, cap, bell (further subdivided into early and late bell), apposition, and eruption stages, based on the histological features of the tooth germ, Figure 2 [20]. The late bell stage marks the onset of dentinogenesis and amelogenesis, while apposition involves the completion of crown formation and the initiation of root development. These sequential stages are conserved across both primary (deciduous) and secondary (permanent) dentitions.

### 3.1. The Bud Stage

**Figure 3:**
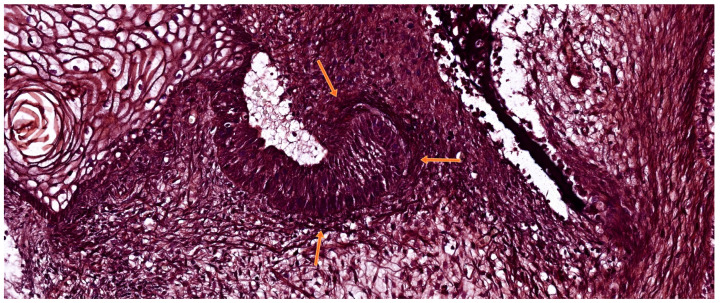
Histological section of a developing human tooth at the bud stage, stained with hematoxylin-eosin (HE). The enamel organ (vertical arrow) appears as a spherical epithelial invagination extending from the oral epithelium. Beneath it, the dental papilla (lower arrow) is seen as a condensed aggregation of ectomesenchymal cells. Surrounding the developing germ, the dental follicle (horizontal arrow) begins to form a mesenchymal capsule. At this stage, the enamel organ has not yet developed distinct epithelial stratification. Scale bar = 100 µm. Original magnification: 10×.

The bud stage marks the initial morphological phase of tooth germ development. It begins when the dental lamina, derived from the oral ectoderm, undergoes localized epithelial proliferation and invaginates into the underlying condensed ectomesenchyme. This results in the formation of a spherical epithelial thickening—the tooth bud—which remains connected to the surface oral epithelium via the dental lamina. As illustrated in Figure 3, this bud penetrates the mesenchymal tissue, initiating the early organization of the enamel organ and dental papilla [21].

This stage is defined by high mitotic activity within the basal epithelial layer, yet cellular differentiation is minimal. The surrounding ectomesenchymal cells condense tightly around the epithelial invagination, setting the foundation for future dental papilla formation. In humans, the bud stage occurs between the 5th and 7th weeks of gestation and gives rise to ten tooth buds in each jaw, corresponding to the future primary dentition [22]. During the bud stage, the developing tooth germ consists of an undifferentiated mass of epithelial cells, with no apparent histological stratification. This stage is characterized by vigorous proliferative activity within the basal layer of the dental lamina, as well as in the adjacent ectomesenchyme. As epithelial proliferation continues, the tooth bud extends deeper into the underlying mesenchyme while remaining tethered to the oral epithelium by a narrow epithelial strand known as the gubernaculum dentis. This structure may later fragment or persist as epithelial cell rests, which can serve as a source for odontogenic cysts in the maxilla [23].

The surrounding ectomesenchymal cells undergo condensation beneath and around the epithelial bud, marking the initiation of the dental papilla, which will later give rise to the dental pulp. A thin basement membrane separates the dental epithelium from the underlying mesenchyme and serves as an essential interface for reciprocal molecular signaling. At this stage, key epithelial signals—including Sonic hedgehog (SHH), Fibroblast growth factors (FGFs), and Bone morphogenetic proteins (BMPs)—begin to orchestrate mesenchymal gene expression and establish the odontogenic field [4].

At this stage, the epithelial cells within the enamel organ remain undifferentiated, retaining a low-columnar or polygonal morphology and exhibiting high mitotic activity. Cytodifferentiation has not yet been initiated. Similarly, the surrounding ectomesenchymal cells remain unspecialized but begin to condense in response to epithelial-derived molecular signals. The adjacent jaw mesenchyme, in contrast, remains more loosely organized [24].

The entire epithelial structure, along with the associated condensed mesenchyme, is referred to as the tooth germ or dental primordium. A hallmark of this stage is the onset of reciprocal epithelial-mesenchymal signaling, which guides the progression toward morphogenesis. Key signaling molecules, including FGFs and SHH, are secreted by the dental epithelium and promote proliferation and apical invagination of the bud. Notably, experimental inhibition of SHH signaling at this stage has been shown to arrest epithelial invagination, highlighting its essential role in bud elongation and morphogenesis [25].

By the end of the bud stage, the number and spatial positioning of individual tooth germs are genetically specified within the developing maxillary and mandibular arches, thereby establishing the blueprint for subsequent morphogenetic patterning during the cap and bell stages.

### 3.2. The Cap Stage

The cap stage is characterized by the enlargement and invagination of the epithelial tooth bud, which begins to fold over the condensed ectomesenchyme, thereby partially enclosing the developing dental papilla. This transition typically occurs between the 8th and 10th weeks of human embryonic development, particularly for the deciduous dentition [26].

**Figure 4:**
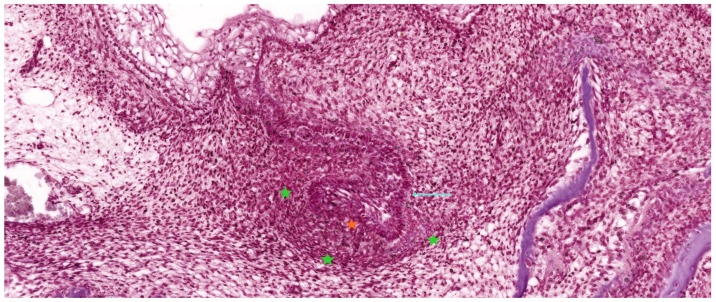
Histological section of a developing human tooth at the early cap stage, stained with hematoxylin–eosin (HE). The enamel organ (cyan arrow) appears as a folded epithelial structure invaginating from the oral epithelium and partially surrounding the underlying dental papilla (orange asterisk). The surrounding ectomesenchyme begins to condense, forming the dental follicle (green asterisks) as a primitive capsule around the developing tooth germ. At this stage, the enamel organ has not yet formed fully distinct layers but displays early compartmentalization. Scale bar = 100 µm. Original magnification: 10×.

**Figure 5:**
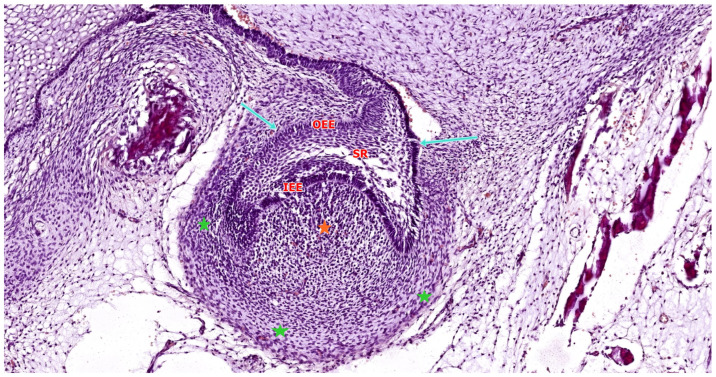
Histological section of a developing human tooth at the late cap stage, stained with hematoxylin–eosin (HE). The enamel organ (cyan arrows) exhibits a cap-like epithelial structure and is compartmentalized into distinct layers: the outer enamel epithelium (OEE), the stellate reticulum (SR), and the inner enamel epithelium (IEE). The underlying dental papilla (orange asterisk) appears as a dense mesenchymal condensation, while the surrounding dental follicle (green asterisks) begins to organize as a vascularized connective tissue capsule. At this stage, the enamel organ starts to reflect early crown morphology. Scale bar = 100 µm. Original magnification: 10×.

As illustrated in Figure 4 and Figure 5, the enamel organ assumes a hemispherical, cap-like morphology with a shallow concavity oriented toward the underlying mesenchymal core. At this stage, the tooth germ becomes histologically compartmentalized into three distinct structures: the enamel organ (epithelial in origin), the dental papilla (mesenchymal condensation that will form dentin and pulp), and the dental follicle (a surrounding mesenchymal sheath that will give rise to the periodontium) [16].

The enamel organ, which represents the epithelial component of the tooth germ, is destined to give rise to the enamel layer of the future tooth. During the early cap stage—illustrated in Figure 5—this structure begins to exhibit internal morphological organization. It differentiates into three distinct epithelial layers: the outer enamel epithelium (OEE), forming the peripheral convex surface; the inner enamel epithelium (IEE), composed of tall columnar cells lining the concave inner surface adjacent to the dental papilla; and the stellate reticulum (SR), a central network of loosely arranged star-shaped cells occupying the space between OEE and IEE. This layered architecture reflects the onset of functional regionalization within the enamel organ and prepares the ground for future cellular differentiation and matrix secretion [24,27].

The enamel organ gradually differentiates into four epithelial layers: the IEE, which harbors progenitor cells that will become ameloblasts; the OEE, serving a protective and structural role; the SR, which forms a hydrated core through glycosaminoglycan secretion; and the stratum intermedium (SI), which supports ameloblast function and enamel mineralization by providing alkaline phosphatase and regulatory molecules. The SR reaches peak development around the 5th–6th months of gestation and later regresses as eruption approaches [28].

As the enamel organ transitions from the bud to the early cap stage, internal differentiation becomes increasingly apparent. Although the SR is not yet fully developed, the enamel organ now displays a hydrated, expanded central region indicative of tissue stratification (Figure 4).

The primary enamel knot (pEK), a transient, non-proliferative epithelial signaling center, first becomes visible during the late bud stage and persists into the early cap stage, where it plays a key role in cusp patterning [29].

This structure serves as a pivotal signaling center that regulates crown morphogenesis. The pEK remains growth-arrested through the expression of cell-cycle inhibitors such as p21 and actively secretes a repertoire of morphogens, including Sonic Hedgehog (SHH), Bone Morphogenetic Proteins (BMP2, BMP4), Fibroblast Growth Factor 4 (FGF4), and Wnt family members. These signals coordinate the proliferation and folding of adjacent epithelial cells and delineate the future cusp pattern of the tooth crown. Following its organizing role, the pEK undergoes programmed apoptosis, and by the late cap stage, it is no longer histologically visible [28].

As the enamel organ enlarges and folds around the condensed mesenchyme within its concavity, a distinct population of mesenchymal cells beneath the inner enamel epithelium undergoes a transition: they reduce their extracellular matrix production and become densely packed. This process leads to the formation of the dental papilla, a mesenchymal condensation that will later differentiate into the odontoblasts and dental pulp. A basement membrane delineates the interface between the IEE and the underlying dental papilla. At this site, bidirectional epithelial–mesenchymal signaling is already underway. Molecular signals from both the primary enamel knot and the IEE, including SHH and BMP4, act upon the papilla mesenchyme to maintain its condensed architecture and initiate odontoblastic fate commitment [29].

Encasing both the enamel organ and dental papilla, the surrounding ectomesenchymal tissue condenses to form the dental follicle (also known as the dental sac). This structure consists of a dense layer of undifferentiated mesenchymal cells that delineate the boundary of the developing tooth germ. Although not yet histologically complex, the dental follicle represents the progenitor tissue for the future cementum, periodontal ligament, and alveolar bone—the principal components of the periodontium [30].

The follicle remains separated from the enamel organ by a basement membrane enveloping the outer enamel epithelium and from the dental papilla by the basal lamina surrounding the papilla’s periphery. Notably, the dental follicle is richly vascularized, supplying essential nutrients and signaling molecules to the avascular enamel organ and dental papilla throughout early odontogenesis [29].

By the end of the late cap stage, the primary architectural components of the tooth germ are well defined, as illustrated in Figure 5. These include the enamel organ, composed of distinct epithelial layers responsible for future enamel formation; the underlying dental papilla, a mesenchymal condensation that will give rise to dentin and pulp; and the surrounding dental follicle, which contributes to the formation of periodontal support structures. At this point, tooth germs also begin to exhibit the earliest features of crown-specific morphology. For example, molar germs display a broader and more voluminous enamel organ, which will subsequently develop multiple enamel knots corresponding to future cusps. In contrast, incisor germs remain smaller and exhibit a simpler morphology consistent with their single-cusp crown pattern [31].

Throughout the cap stage, reciprocal signaling between the dental epithelium and the underlying mesenchyme remains highly active. Under the influence of epithelial signals, the dental papilla mesenchyme begins to express key transcription factors such as PAX9 and MSX1, which are essential for proper tooth morphogenesis [29]. These mesenchymal factors, in turn, act upstream of several epithelial signaling cascades, contributing to the progression and patterning of the enamel organ.

Experimental studies in mouse models have demonstrated that Msx1 deficiency results in failure of the tooth germ to progress beyond the bud or cap stage. This arrest is associated with downregulation of essential epithelial signals, including Bmp4 and Fgf3, highlighting the importance of mesenchymal input in sustaining epithelial morphogenesis [32].

Thus, the cap stage represents a critical developmental checkpoint where bidirectional signaling—emanating from the enamel knot and inner enamel epithelium and reciprocated by the condensed dental papilla—drives the increasing structural and functional complexity of the developing tooth germ.

### 3.3. The Bell Stage

As the enamel organ continues to proliferate and undergo morphological folding, the developing tooth germ transitions into the bell stage—a critical phase characterized by advanced tissue organization and early functional commitment. This stage is classically divided into two subphases: the early bell stage, during which morphodifferentiation is completed but matrix secretion has not yet commenced, and the late bell stage, marked by the onset of dentinogenesis and amelogenesis [32]. In human embryonic development, the bell stage typically spans the 11th to 14th weeks of gestation, encompassing much of the second trimester as the crowns of deciduous teeth take shape.

#### 3.3.1. Early Bell Stage

**Figure 6:**
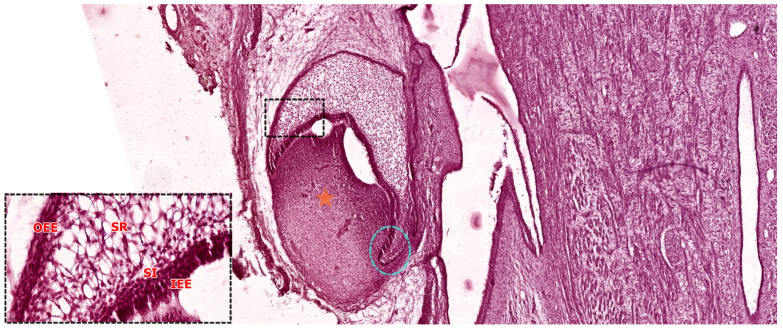
Histological section of a developing human tooth at the early bell stage, stained with hematoxylin-eosin (HE). The enamel organ exhibits a bell-shaped morphology and is composed of four epithelial layers: the outer enamel epithelium (OEE), stellate reticulum (SR), stratum intermedium (SI), and inner enamel epithelium (IEE), highlighted in the high-magnification inset (original magnification: 40×, scale bar = 100 µm). The cervical loop (dotted circle) marks the junction between the outer and inner enamel epithelium and serves as a proliferative zone that contributes to both crown and root development. The dental papilla (orange asterisk) is seen beneath the IEE as a densely packed mesenchymal condensation that will differentiate into odontoblasts and dental pulp. Scale bar (main image) = 100 µm. Original magnification: 10×.

During the early bell stage, the enamel organ adopts a bell-like morphology, characterized by the presence of four distinct epithelial layers: the OEE, the SR, the SI, and the IEE. As shown in Figure 6, this stage reflects a high degree of histological compartmentalization and preparation for secretory differentiation [33].

The OEE, located at the convex periphery of the enamel organ, is composed of a layer of cuboidal or low columnar cells that play a mechanical and protective role. Beneath it, the stellate reticulum consists of loosely connected, star-shaped cells embedded in an extracellular matrix rich in glycosaminoglycans. These hydrophilic molecules retain water, contributing to internal hydrostatic pressure and tissue expansion, while also facilitating calcium diffusion necessary for future enamel mineralization [24]. The enamel organ remains connected to the oral epithelium through the gubernaculum dentis, a cellular cord derived from the dental lamina. This structure will undergo regression during the late bell stage.

In the early bell stage, the SI becomes more prominent, forming a 4–5 cell-thick layer of cuboidal cells situated between the stellate reticulum and the inner enamel epithelium. These cells display high enzymatic activity, notably alkaline phosphatase, and function cooperatively with ameloblasts to support enamel matrix mineralization and cellular maturation [26].

The IEE, composed of columnar cells lining the concave inner surface of the enamel organ, plays a pivotal inductive role in the development of the underlying dental papilla. At the junction between the inner enamel epithelium IEE and outer enamel epithelium OEE, the cervical loop marks an area of intense epithelial proliferation. This structure generates a continuous supply of ameloblast precursors during crown formation and will later extend apically into the mesenchyme to give rise to Hertwig’s epithelial root sheath (HERS), a bilayered epithelium that governs root morphogenesis [34].

Within the dental papilla—of ectomesenchymal origin—two zones can be distinguished. The central zone is cell-rich and will give rise to the dental pulp, while the peripheral zone, adjacent to the IEE, is defined by a condensed population of mesenchymal cells and collagen fibers, including the von Korff fibers. These fibrils, part of Retterer’s hematoxylinophilic network, are among the earliest indicators of dentinogenesis [35].

The basement membrane that separates the IEE from the dental papilla gradually fragments at this stage, permitting epithelial–mesenchymal contact. This interaction initiates the differentiation of peripheral papilla cells into odontoblasts, marking the transition to the secretory phase of dentinogenesis, after which the papilla becomes structurally and functionally defined as the dental pulp.

In addition to structural organization, the early bell stage is characterized by distinct immunohistochemical profiles that define both epithelial and mesenchymal compartments. Distinct immunohistochemical signatures characterize the epithelial and mesenchymal components of the developing tooth germ. In the enamel organ, most epithelial cells express cytokeratin 14 (CK14), except for preameloblasts and secretory ameloblasts, which are positive for CK19. The stellate reticulum (SR) exhibits CK7 expression, while the outer enamel epithelium occasionally co-expresses CK13 [27].

Cells of Hertwig’s epithelial root sheath (HERS) also express CK7, and both the inner and outer enamel epithelium begin to show markers of mesenchymal-type differentiation in the late bell stage, such as vimentin and glial fibrillary acidic protein (GFAP). SR cells additionally demonstrate immunoreactivity to smooth muscle actin (SMA), suggesting a transient contractile phenotype [36].

A subset of enamel organ cells displays immunopositivity for CD117 (c-Kit), indicative of stem/progenitor potential. p63, a transcription factor associated with epithelial renewal and stratification, is also robustly expressed across all enamel organ layers. E-cadherins, critical for cell adhesion, are consistently observed during both the cap and bell stages [37].

Within the dental papilla, cells express vimentin and actin, consistent with their mesenchymal origin. Stem cells within the papilla show positivity for CD133, CD117, and HLA-DR and exhibit multipotent potential, expressing lineage-specific markers such as: adipogenic (leptin, adipophilin), myogenic (desmin, myogenin, myosin II, SMA), neurogenic (gamma-enolase, nestin, GFAP, βIII-tubulin), osteogenic (osteonectin, osteocalcin, osteopontin, collagen I), and chondrogenic (collagen II, SOX9) [38].

Moreover, cells of the dental papilla exhibit strong expression of angiogenic and growth signaling molecules, including VEGF, VEGFR2, EGF, and EGFR, underscoring the metabolic and regenerative potential of this compartment during early tooth development [39].

#### 3.3.2. Late Bell Stage

**Figure 7:**
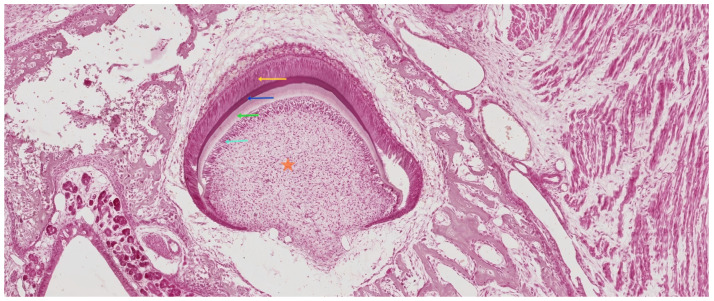
Histological section of a developing human tooth at the late bell stage, stained with hematoxylin–eosin (HE). The dental papilla (orange asterisk), now transitioning into the dental pulp, is bordered peripherally by a single layer of odontoblasts (cyan arrow), responsible for secreting the dentin matrix (green arrow). Above this, the mineralizing enamel layer (blue arrow) is secreted by the overlying ameloblasts (yellow arrow), which are elongated, polarized epithelial cells derived from the inner enamel epithelium. At this stage, the enamel organ is fully developed and functionally compartmentalized. Scale bar = 100 µm. Original magnification: 10×.

The late bell stage marks the onset of crown formation, characterized by the initial deposition of dentin and enamel matrices. These events are driven by the differentiation and secretory activity of odontoblasts and ameloblasts, respectively [18]. As illustrated in Figure 7, this stage signifies the functional transition of the enamel organ and dental papilla into active matrix-producing tissues.

In parallel with enamel organ maturation, the dental papilla undergoes proliferation and histodifferentiation within the concavity of the enamel organ. At its periphery, under the inductive influence of preameloblasts—derived from the inner enamel epithelium—mesenchymal precursor cells differentiate into preodontoblasts and subsequently into secretory odontoblasts. These odontoblasts localize initially at the future cusp tip, where they begin to secrete the first increment of organic matrix, known as predentin. This unmineralized layer undergoes progressive mineralization, giving rise to the earliest coronal dentin [40].

Dentinogenesis precedes amelogenesis both spatially and temporally. Once a sufficient dentin matrix is deposited, ameloblasts initiate their secretory activity, laying down enamel matrix directly atop the mineralized dentin surface. Notably, enamel matrix mineralizes at a rate approximately twice as fast as that of dentin, reflecting its unique physicochemical composition and developmental dynamics [41].

##### Differentiation of Odontoblasts

The differentiation of odontoblasts is initiated by inductive signals from preameloblasts, which arise from the IEE. Prior to the onset of dentinogenesis, IEE cells elongate, adopt a columnar morphology, and align along the basement membrane that separates the enamel organ from the underlying dental papilla. This morphogenetic rearrangement coincides with intense proliferative activity in the IEE and reflects their increasing functional polarization [42].

At this stage, a transient acellular zone becomes evident between the IEE and the peripheral dental papilla. This region is devoid of differentiated cells and serves as a permissive interface for epithelial–mesenchymal interaction. The adjacent mesenchymal cells of the papilla are small, undifferentiated, and characterized by a centrally located nucleus and sparse cytoplasmic organelles. These ectomesenchymal cells represent odontogenic progenitors, which will soon undergo polarization and elongation, forming the first layer of preodontoblasts at the interface with the IEE [43].

As preameloblasts complete their morphological transformation, cell division within the IEE ceases. The IEE cells become columnar and polarize, with the nucleus migrating away from the basement membrane, establishing apico-basal polarity [44]. This epithelial change serves as a critical trigger for odontoblast induction.

Adjacent to the acellular zone, ectomesenchymal cells of the dental papilla increase in size and begin to polarize, forming preodontoblasts. These cells display prominent endoplasmic reticulum and Golgi apparatus, consistent with their impending secretory role. Preodontoblasts express receptors for cytokines such as interleukin-6 (IL-6) and interleukin-10 (IL-10), which may modulate their responsiveness to inductive cues during differentiation [45,46].

As odontoblasts mature, the acellular zone progressively disappears, being replaced by a single layer of elongated, fully differentiated odontoblasts. These cells align along the basement membrane, which not only maintains structural integrity at the epithelial–mesenchymal interface but also contributes bioactive matrix components that guide odontoblast polarization and positioning [42].

During the final mitotic events in the odontogenic mesenchyme, the mitotic spindle of dividing papilla cells aligns perpendicular to the basement membrane, resulting in vertically superimposed daughter cells. Only the basal cell, in direct contact with the membrane, receives sufficient inductive signals to differentiate into an odontoblast. The more superficial daughter cell remains undifferentiated, giving rise to the subodontoblastic population, also known as Höhl cells [3,41,47]. A summary of the sequential events, molecular features, and functional outcomes associated with odontoblast differentiation is provided in Table 2.

In physiological conditions, this spatial asymmetry ensures the formation of a single, polarized layer of odontoblasts. However, under reparative conditions, such as after injury, subodontoblastic cells can serve as a reservoir of progenitors, capable of differentiating into new odontoblasts in the absence of epithelial cues. Odontoblast differentiation depends not only on epithelial contact but also on the composition of the surrounding extracellular matrix (ECM). The basement membrane undergoes partial fragmentation during this phase, facilitating direct epithelial–mesenchymal interactions. This membrane contains growth factors and proteoglycans that modulate signaling gradients and cell polarization [49].

Among the key ECM components, fibronectin plays a dual role as a structural matrix protein and cell adhesion molecule, guiding odontoblast alignment and elongation. In contrast, tenascin, though not associated with the basement membrane, is highly expressed in regions of mesenchymal condensation during early tooth development and contributes to patterning the odontogenic field [50].

##### Differentiation of Ameloblasts

The transformation of inner enamel epithelial cells into secretory ameloblasts is initiated by inductive cues—primarily from underlying odontoblasts—which trigger cytoskeletal reorganization and cellular polarization. Microtubules and actin filaments contribute to the alignment and elongation of these cells, which first appear as preameloblasts [48].

As differentiation progresses, preameloblasts elongate further and acquire the molecular and ultrastructural features of mature ameloblasts. These columnar cells exhibit apico-basal polarity, with the nucleus located basally and the secretory pole oriented toward the dentin surface. The cytoplasm becomes enriched in secretory organelles, including mitochondria, rough endoplasmic reticulum, and a prominent Golgi apparatus, reflecting high protein synthetic activity [51].

At the apical pole, ameloblasts develop a conical cytoplasmic extension known as Tomes’ process, which is essential for the structured deposition of enamel prisms. This process contains numerous secretory vesicles packed with enamel-specific proteins such as amelogenins and enamelins. Tight junctions between adjacent ameloblasts—both apically and basally—help form the internal and external ameloblastic membranes, which maintain structural cohesion and regulate ion and protein flow during enamel biomineralization [52].

### 3.4. The Crown Stage—Apposition

The apposition stage marks the coordinated deposition of the dentin and enamel matrices, representing the final phase of crown morphogenesis. At this point, odontoblasts and ameloblasts are fully differentiated and engaged in polarized secretion of their respective extracellular matrices. Dentin is deposited incrementally by odontoblasts, while ameloblasts simultaneously initiate enamel matrix secretion at the dentin–enamel junction. Once enamel deposition is completed, the enamel organ collapses, and its cellular components reorganize to form the reduced enamel epithelium—a flattened bilayer derived from the inner, outer, and intermediate epithelial layers. This epithelial covering protects the mature enamel surface and will later fuse with the oral epithelium to guide the eruption pathway of the tooth [53].

**Figure 8:**
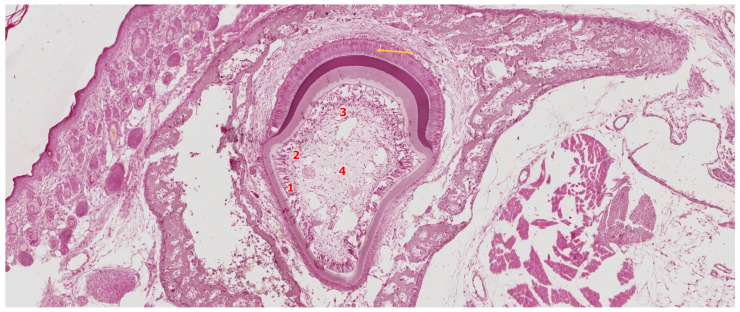
Histological section of a developing human tooth at the apposition stage, stained with hematoxylin–eosin (HE). The dental pulp is fully organized and displays four distinct zones: (1) a peripheral layer of secretory odontoblasts, (2) the acellular zone of Weil, (3) a densely populated cell-rich zone, and (4) a central zone containing blood vessels and nerve fibers. Adjacent to the odontoblast layer, predentin is secreted and progressively mineralized into dentin. Overlying this, the enamel matrix is deposited by tall, polarized secretory ameloblasts (yellow arrow), indicating active amelogenesis. Scale bar = 100 µm. Original magnification: 10×.

The apposition stage marks the coordinated deposition of the dentin and enamel matrices, representing the final phase of crown morphogenesis. At this point, odontoblasts and ameloblasts are fully differentiated and engaged in polarized secretion of their respective extracellular matrices. Dentin is deposited incrementally by odontoblasts, while ameloblasts simultaneously initiate enamel matrix secretion at the dentin–enamel junction. As shown in Figure 8, histological sections at this stage reveal the spatial organization of the developing crown, with distinct tissue zones including the predentin–dentin complex, active ameloblasts, and the structured dental pulp.

The deposition of dentin and enamel during the apposition stage is tightly synchronized, ensuring the proper formation of the dentino–enamel junction (DEJ)—a critical interface that provides both mechanical integrity and developmental coordination. Dentinogenesis always precedes amelogenesis, with odontoblast activity acting as a prerequisite for the induction and function of overlying ameloblasts. The enamel matrix is secreted precisely upon the mineralized dentin front, and its structural organization is guided by the underlying tubular pattern of dentin. This spatial and temporal coordination between mesenchymal and epithelial compartments reflects a conserved developmental mechanism that ensures crown integrity and prepares the cervical loop for its subsequent role in root morphogenesis [54].

### 3.5. Root Formation

Root development is initiated only after the crown has reached its final morphology. The process begins at the cervical loop, where the inner and outer enamel epithelia proliferate apically to form Hertwig’s epithelial root sheath—a bilayered epithelial structure responsible for guiding root elongation and patterning. As HERS extends downward around the base of the dental papilla, it defines the shape and length of the developing root. Its apical termination bends inward to form the epithelial diaphragm, delimiting the primary apical foramen and separating the dental papilla mesenchyme from the surrounding dental follicle [55].

Under the inductive influence of the inner epithelial layer of HERS, peripheral cells of the papilla differentiate into radicular odontoblasts, initiating radicular dentinogenesis. As dentin is deposited apically and centrifugally, HERS undergoes fragmentation, exposing areas of newly formed dentin to the follicular mesenchyme. This fragmentation permits the differentiation of cementoblasts from the inner follicular zone, which initiate cementum formation. Simultaneously, osteoblasts arising from the outer follicular zone contribute to the formation of alveolar bone, while the remaining connective tissue organizes into the periodontal ligament [30].

In some cases, remnants of HERS persist as isolated epithelial clusters in the periodontal ligament, known as the epithelial cell rests of Malassez. These may serve as a source for odontogenic cysts or tumors under pathological conditions [56]. Although radicular odontoblasts differentiate via mechanisms analogous to those in the crown, they are not preceded by preameloblast induction. Instead, they are directly influenced by HERS epithelium. These cells polarize, align along the basement membrane, and secrete predentin, which mineralizes centripetally. This process continues until root elongation is completed—typically around one year after tooth eruption [57].

Radicular dentin differs structurally from coronal dentin: it contains less phosphorin, exhibits lower mineral density, and lacks a distinct mantle dentin layer. The collagen fibers within radicular dentin are oriented parallel to the long axis of the root, contributing to its unique biomechanical properties [58].

## 4. Development of Periodontal Support Tissues

The periodontium comprises the cementum, periodontal ligament (PDL), alveolar bone, and gingiva—tissues that collectively support and anchor the tooth within the alveolar process. These structures originate from the dental follicle, also known as the dental sac, a condensed ectomesenchymal tissue that surrounds the enamel organ and dental papilla beginning in the bell stage [48].

As root formation progresses, the dental follicle gives rise to multiple specialized cell lineages: cementoblasts, which form the cementum layer over radicular dentin; fibroblasts, which synthesize the collagen-rich periodontal ligament; and osteoblasts, which contribute to alveolar bone formation. The gingiva, although closely associated with the periodontium, develops later, following tooth eruption, as part of the integration between the reduced enamel epithelium and oral mucosa [59].

### 4.1. Cementogenesis

The formation of cementum is initiated following fragmentation of HERS, which exposes the outer surface of newly deposited radicular dentin. Cells from the dental follicle, upon contact with this surface, differentiate into cementoblasts. These cells exhibit features characteristic of secretory function, including a well-developed rough endoplasmic reticulum and Golgi apparatus [36].

Prior to complete fragmentation of HERS, a thin, highly mineralized layer is formed over the radicular dentin, historically referred to as Hopewell-Smith’s hyaline layer, or intermediate cementum. This layer originates from epithelial remnants and acts as an anchoring interface between dentin and cementum [60].

Cementoblasts then secrete an organic matrix composed primarily of collagen fibers and ground substance, known as cementoid. Mineralization of this matrix proceeds via hydroxyapatite crystal deposition, closely resembling the mineralization process of dentin. The initial cementum formed is typically acellular, as cementoblasts remain at the periphery and retreat into the forming PDL [61].

Cementum is deposited incrementally in successive lamellae, oriented parallel to the root surface, with growth lines demarcating periods of activity. The thickness of cementum increases with age, reaching its maximum at the apical third of the root, particularly near the apical foramen. Although cementogenesis slows after tooth eruption, the process remains intermittently active throughout life, supporting tooth stability and adaptation to occlusal forces [62].

### 4.2. Degeneration of Hertwig’s Root Sheath

Following completion of root dentinogenesis, Hertwig’s epithelial root sheath (HERS) undergoes fragmentation and involution. As this process progresses, epithelial remnants of HERS become dispersed within the developing PDL. These epithelial cell clusters, known as the epithelial cell rests of Malassez (ERM), persist adjacent to the cementum surface, embedded within the ligament matrix [30]. Histologically, ERM appears as small islands of quiescent epithelial cells arranged in a loosely connected network. Although their number tends to decline with age, many of these cells persist into adulthood and are thought to contribute to PDL homeostasis, including regulation of repair, regeneration, and response to mechanical stress [63].

Under pathological conditions, particularly in response to chronic inflammation or tissue injury, ERM may become reactivated, proliferate, and participate in the formation of odontogenic cysts or, more rarely, benign or malignant tumors. This pathological transformation is often associated with epithelial cavitation, leading to the formation of epithelial-lined cystic structures within the periodontal tissues [64].

### 4.3. Formation of the Periodontal Ligament

The PDL begins to form shortly after the initiation of root development, as cells from the dental follicle differentiate into PDL fibroblasts. These fibroblasts rapidly proliferate and acquire a collagen-synthesizing phenotype, contributing to the formation of collagen fiber bundles that span between the developing cementum and alveolar bone [61].

Prior to tooth eruption, the alveolar crest lies coronal to the cementoenamel junction (CEJ), and the developing fiber bundles are oriented obliquely. As the tooth erupts and advances occlusally, the position of the alveolar bone adjusts accordingly. Fiber orientation shifts, with bundles realigning horizontally and, eventually, obliquely apically, once the tooth reaches its functional position [65].

The PDL remains in a state of active remodeling throughout development and adult life. This dynamic remodeling is regulated primarily by fibroblasts, which exhibit both synthetic (collagen precursors) and degradative (collagenase) activities. This dual role ensures tissue renewal, adaptation to occlusal forces, and the maintenance of periodontal integrity [66].

### 4.4. Formation of the Alveolar Bone

The alveolar bone develops from the ectomesenchymal cells of the dental follicle, initially forming at the periphery of the developing periodontal ligament. Its formation begins around the 8th week of intrauterine life, in parallel with tooth organogenesis. Early in development, a bony crypt surrounds each dental germ, providing mechanical support and containing the neurovascular bundle that supplies the forming tooth [67].

As development proceeds, the alveolar process expands through appositional bone growth, and its structure becomes compartmentalized into the alveolar bone proper and supporting bone. During the period of rapid growth, especially at the alveolar crests, a transitional tissue with features intermediate between cartilage and bone—termed chondroid bone—may transiently appear, contributing to the adaptability of the developing maxilla and mandible [68].

The eruption of permanent teeth is preceded by physiological resorption of the overlying alveolar bone and the roots of primary teeth, a process that is more efficient in deciduous dentition. After tooth loss, the alveolar ridge undergoes progressive vertical and horizontal resorption, leading to a reduction in bone height and width—a phenomenon with significant clinical implications in prosthodontics and implantology [69].

### 4.5. Development of the Dento-Gingival Junction

The formation of the dento-gingival junction begins during the late stages of crown formation, when the tooth is fully covered by the reduced enamel epithelium (REE). This bilayered structure is derived from the inner enamel epithelium-now composed of reduced ameloblasts and remnants of the outer enamel epithelium and stratum intermedium. REE cells establish adhesion to the enamel surface via hemidesmosomes and a specialized basement membrane. During tooth eruption, the REE approaches and fuses with the oral epithelium, forming a continuous epithelial mass over the erupting crown. This fusion is accompanied by degradation of the intervening connective tissue, leading to epithelial remodeling and formation of a non-hemorrhagic eruption pathway [70].

Following emergence into the oral cavity, basal cells from the oral epithelium migrate apically along the enamel surface. Together with cells derived from the REE, they contribute to the formation of the JE, which provides adhesive and immunological protection at the tooth–gingiva interface [71]. Over time, the REE transforms into stratified squamous epithelium, but this transition occurs gradually and is not fully complete until 3–4 years post-eruption. During this maturation period, the gingival sulcus is formed, and the JE remains attached to the enamel via hemidesmosomes and basal lamina [24].

The apical extent of the JE defines the final position of the dento-gingival junction, which normally reaches the CEJ. However, with aging, inflammation, or surgical procedures (e.g., gingivectomy), apical migration of the junction can occur—a phenomenon referred to as passive eruption—resulting in clinical crown elongation and potential periodontal exposure [72].

Importantly, the junctional epithelium can regenerate after injury or surgical removal, primarily through proliferation of oral epithelial stem cells, supported by the underlying connective tissue. This regenerative capacity plays a critical role in maintaining the structural and functional integrity of the gingival seal throughout life.

Developmental dental anomalies not only include variations in tooth number, size, and shape but also defects in the structure and composition of dental tissues. Among these, amelogenesis imperfecta and dentinogenesis imperfecta are two well-characterized hereditary conditions that impact enamel and dentin formation, respectively. Amelogenesis imperfecta encompasses a group of disorders affecting enamel thickness and mineralization, often caused by mutations in AMELX, ENAM, or MMP20 genes. Patients present with enamel that is thin, hypoplastic, or hypomineralized, resulting in significant aesthetic and functional impairments [73]. On the other hand, dentinogenesis imperfecta, frequently associated with DSPP gene mutations, results in opalescent, discolored teeth with structurally weak dentin, prone to rapid wear and pulpal obliteration. These conditions highlight the clinical importance of understanding molecular mechanisms underlying enamel and dentin formation, and they provide a direct link between developmental biology and clinical dentistry.

## 5. Materials and Methods

The histological images included in this review derive from archived human embryonic tissue slides processed in the Department of Histology at the “Victor Babeș” University of Medicine and Pharmacy, Timișoara. These samples were collected more than a decade ago, during routine academic dissection and slide preparation for teaching purposes.

Tissue specimens were fixed in 10% neutral buffered formalin, embedded in paraffin, and sectioned at 4–6 µm using a standard rotary microtome. The sections were mounted on glass slides and stained with hematoxylin and eosin (H&E) using traditional manual protocols.

All images were captured using a brightfield microscope equipped with a digital camera at original magnifications of 10× or 40×, as indicated in figure legends. Minor variations in staining intensity reflect historical differences in preparation and storage. No digital post-processing was applied to preserve the authenticity of the original material.

## 6. Molecular Regulation of Odontogenesis

In the earliest stages, particularly around embryonic day 10, signaling molecules such as bone morphogenetic proteins (BMPs) and fibroblast growth factors (FGFs) are expressed in the oral epithelium. The interplay between BMP-4 and FGF-8 establishes the boundaries of odontogenic competence in the underlying mesenchyme by inducing genes such as Pax9, Msx1, and Msx2. This coordinated epithelial signaling defines the sites at which dental placodes will emerge and dictates future tooth identity [74].

By day 11 of intrauterine development, additional BMPs (BMP-2, BMP-7) and FGFs (FGF-9) are also expressed in the dental epithelium, reinforcing mesenchymal gene expression programs. The mesenchyme, in response, begins to express its own set of signaling molecules, including BMP-4, FGF-3, and activin βA, signifying a shift in odontogenic potential from epithelium to mesenchyme. This bidirectional exchange marks a pivotal transition point in the bud stage of odontogenesis and is critical for further morphogenesis [75].

During the cap stage, these signaling interactions intensify and become spatially localized. The primary enamel knot (pEK), a transient non-proliferative signaling center within the inner enamel epithelium, secretes key morphogens such as Sonic Hedgehog (SHH), BMP2/4, FGF4, and specific Wnt ligands. Among these, Wnt10a and Wnt10b are highly expressed in the enamel knot and regulate epithelial folding and cusp patterning via the canonical Wnt/β-catenin pathway. Additionally, Wnt5a, expressed in adjacent mesenchymal cells, contributes to non-canonical signaling that modulates cell migration and orientation. These pathways together control proliferation, differentiation, and apoptosis in surrounding tissues, ensuring proper morphogenesis of the tooth crown [6,76].

FGF signaling continues to play a crucial role beyond initiation. FGF-8 contributes to Barx1 induction in molar mesenchyme, while FGF-9 and FGF-4 stimulate proliferation in both epithelial and mesenchymal compartments. Notably, FGF-10 promotes proliferation only in the epithelium, underscoring ligand-specific roles within the FGF family. Collectively, these pathways coordinate the size, shape, and cusp architecture of the developing tooth [77].

A number of transcription factors act downstream of these signaling cues to guide morphogenesis and cytodifferentiation. Pax9 is restricted to odontogenic mesenchyme by the combined actions of BMP and FGF signaling and is essential for proper progression beyond the bud stage. Similarly, Msx1 and Msx2 are expressed in the dental papilla and dental follicle throughout the bud, cap, and bell stages. These genes are involved in maintaining mesenchymal cell proliferation and competence to respond to epithelial signals. Additional transcription factors such as Dlx1/2, Lhx6/7, and Pitx2 exhibit distinct expression patterns, particularly within the posterior dental mesenchyme (e.g., molar regions), and contribute to the regional specification of tooth types [78].

These transcriptional regulators not only mediate the cellular response to external signaling but also modulate upstream pathways through feedback loops, ensuring developmental robustness. For instance, Msx1-null mice exhibit arrested tooth development at the bud stage due to loss of Bmp4 expression, underscoring the critical feedback role of mesenchymal transcription factors in sustaining epithelial signaling [79].

Taken together, the regulation of odontogenesis exemplifies a highly integrated system in which signaling gradients, transcriptional hierarchies, and tissue-tissue communication converge to control a morphogenetic sequence. Understanding these molecular frameworks has profound implications—not only for the pathogenesis of congenital dental anomalies such as hypodontia or amelogenesis imperfecta, but also for regenerative strategies aiming to bioengineer replacement teeth from stem or progenitor cells.

## 7. Conclusions

Tooth development is a highly coordinated process that exemplifies the complexity of epithelial–mesenchymal interactions in organogenesis. From the initial formation of the dental lamina to the terminal differentiation of hard tissue-producing cells, odontogenesis involves a sequential interplay of morphogenetic events, histological compartmentalization, and molecular signaling. By integrating classical histological observations with advances in molecular biology, this review highlights the intricate regulatory networks—particularly those involving BMP, FGF, Wnt, and SHH pathways—that govern tissue patterning and cell fate determination throughout the bud, cap, bell, and root formation stages.

The integration of histological and molecular insights into odontogenesis not only enhances academic understanding but also holds significant clinical relevance. These findings contribute to the improvement of diagnostic precision in developmental dental anomalies, inform the design of biomimetic materials for restorative dentistry, and guide regenerative strategies, such as stem cell-based tooth reconstruction. In addition, understanding the cellular signaling pathways that regulate enamel and dentin formation provides a foundation for developing targeted therapies in cases of hypomineralization, amelogenesis or dentinogenesis imperfecta, and periodontal regeneration.

A deeper understanding of these molecular frameworks not only enhances our insight into the pathogenesis of congenital dental anomalies but also opens promising avenues for regenerative therapies, including bioengineered tooth replacement strategies. Future studies should continue to explore the temporal-spatial dynamics of these pathways and their potential manipulation in therapeutic contexts.

## Figures and Tables

**Table 1:** Chronological timeline and origin of human tooth development.

Tooth Type	Developmental Origin	Initiation Time	Notes	Reference
Mandibular Incisors	Primary dental lamina	Week 7 of gestation	First tooth germs to initiate	Hovorakova et al., 2018 [16]
Maxillary Incisors	Primary dental lamina	Week 8 of gestation		Hovorakova et al., 2018 [16]
Canines	Primary dental lamina	Week 8–9 of gestation		Järvinen et al., 2009 [18]
First Primary Molars	Primary dental lamina	Week 8–9 of gestation		Jussila & Thesleff, 2012 [6]
Second Primary Molars	Primary dental lamina	Week 10–11 of gestation		Hovorakova et al., 2018 [16]
Successional Teeth	Successional dental lamina	5th prenatal month (incisors, canines) 10th prenatal month (2nd premolars)	Replace primary teeth	Järvinen et al., 2009 [18]
First Permanent Molar	Posterior extension of primary lamina	~4th month in utero	Accessional tooth (no primary precursor)	Jussila & Thesleff, 2012 [6]
Second Permanent Molar	Posterior extension of primary lamina	1st postnatal year	Accessional	Kwon & Jiang, 2018 [13]
Third Molar (Wisdom)	Posterior extension of primary lamina	Age 4–5 years	Accessional, latest to form	Jussila & Thesleff, 2012 [6]

**Table 2:** Sequential cellular events, molecular characteristics, and functional outcomes involved in odontoblast differentiation during the bell stage of tooth development. Abbreviations: IEE—Inner enamel epithelium; BM—Basement membrane; ER—Endoplasmic reticulum; IL-6/IL-10—Interleukin-6 / Interleukin-10; ECM—Extracellular matrix.

Stage	Cellular/Molecular Features	Functional Outcome	Reference
IEE polarization	Columnar alignment, nuclear shift, cessation of division	Triggers odontoblast induction	Chang et al., 2019 [44]
Acellular zone formation	Space between IEE and papilla	Permissive site for epithelial–mesenchymal signaling	Bleicher et al., 2015 [45]
Preodontoblast emergence	Cell enlargement, ER and Golgi development; IL-6/IL-10 receptor expression	Preparation for dentin matrix secretion	Pan et al., 2023 [48]
Odontoblast differentiation	Basement membrane contact; elongation and alignment	Initiation of predentin secretion	Bleicher et al., 2015 [45]
Mitotic orientation (90° to BM)	Superimposed daughter cells	Basal cell → odontoblast; superficial → Höhl cell	Chang et al., 2019 [44]
Subodontoblast (Höhl cell) reserve	Not induced; remains undifferentiated	Stem/progenitor pool for repair/regeneration	Tziafas & Kodonas, 2010 [23]
Role of basement membrane	Fragmented; enriched with proteoglycans and growth factors	Modulates cell polarity and ECM signaling	Xu et al., 2009 [30]
Key ECM molecules	Fibronectin—adhesion and alignment; Tenascin—mesenchymal patterning	Supports odontoblast organization and morphogenesis	Dalton & Lemmon, 2021 [49]
